# Epidemiological characteristics of a COVID-19 outbreak caused by religious activities in Daegu, Korea

**DOI:** 10.4178/epih.e2021024

**Published:** 2021-04-14

**Authors:** Jong-Yeon Kim, Yu-Mi Lee, Hwajin Lee, Jung-Whan Kim, Shin-Woo Kim

**Affiliations:** 1 Department of Public Health, Kyungpook National University Hospital, Daegu, Korea; 2 Department of Preventive Medicine, School of Medicine, Kyungpook National University, Daegu, Korea; 3 Department of Internal Medicine, School of Medicine, Kyungpook National University, Daegu, Korea

**Keywords:** Coronavirus, COVID-19, Disease outbreaks, Epidemiologic surveillance, Religion, Korea

## Abstract

**OBJECTIVES:**

A coronavirus disease 2019 (COVID-19) outbreak triggered by religious activities occurred in Daegu, Korea in February 2020. This outbreak spread rapidly to the community through high-risk groups. This study describes the characteristics of COVID-19 cases based on S religious group membership and summarizes the Daegu municipal government’s processes and responses to control the outbreak.

**METHODS:**

The epidemiological characteristics of confirmed cases were obtained through basic and in-depth epidemiological surveys. General characteristics, the proportion of asymptomatic cases, the case-fatality rate, and the time-to-event within each group were presented after stratifying confirmed cases according to S religious group membership.

**RESULTS:**

Overall, 7,008 COVID-19 cases were confirmed in Daegu from February 18, 2020 to June 30, 2020, and 61.5% (n=4,309) were S religious group members. Compared with non-members, members had a higher proportion of female (p<0.001) and younger age (p<0.001), as well as lower disease prevalence. At the time of the investigation, 38.4% of cases in members were asymptomatic versus 23.7% of cases in non-members (p<0.001). The case-fatality rate of non-members aged ≥ 60 years was significantly higher than that of members (p<0.001). Compared with non-members, members had longer intervals from symptom onset to diagnosis (p<0.001) and from diagnosis to admission (p<0.001), and a shorter interval from admission to discharge (p<0.001).

**CONCLUSIONS:**

The epidemiological features of S religious group members, including the proportion of asymptomatic cases, case-fatality rate, and time-to-event, differed from non-members. The Daegu authorities prevented further COVID-19 spread through immediate isolation and active screening tests of all S religious group members.

## INTRODUCTION

Coronavirus disease 2019 (COVID-19) was first reported as viral pneumonia in Wuhan, China in December 2019. It then became a pandemic in which > 100 million people were infected by the end of January 2021 [1]. The International Committee on Taxonomy of Viruses *Coronaviridae* Study Group named the causative virus severe acute respiratory syndrome coronavirus 2 (SARS-CoV-2) [2]. With the rapid spread of COVID-19 in high-risk groups from mid-February to early March 2020, the number of confirmed cases in Korea ranked second in the world after China. By the end of January 2021, > 70,000 COVID-19 cases had been confirmed in Korea [3].

It is noteworthy that the COVID-19 outbreak in Korea started mainly from a religious group’s activities in Daegu, a metropolitan city. The 31st confirmed case (case #31) in Korea lived in Daegu and was diagnosed on February 18, 2020 [4]. An epidemiological investigation identified that case #31 was a member of the S religious group, who attended worship services on February 9, 2020 and February 16, 2020 while symptomatic. An investigation was conducted among S religious group members who attended these services (n=1,001). The number of confirmed cases increased, exceeding 2,000 at the end of February 2020 and 5,000 by early March 2020. Most of the confirmed cases were related to the S religious group. As of March 31, 2020, 63.7% (4,257 of 6,684) of all confirmed cases were members of the S religious group [4].

The epidemiological and clinical characteristics of confirmed COVID-19 cases admitted to a tertiary hospital in Daegu (n=694) [5] and the actions of the Daegu municipal health authorities to combat this COVID-19 outbreak [6] have been described. This study analyzed the characteristics of confirmed COVID-19 cases among S religious group members and non-members based on epidemiological surveys. In addition, this study aimed to summarize the processes and responses to the Daegu COVID-19 outbreak, which was caused mainly by religious activities.

## MATERIALS AND METHODS

### Source of data

Information on the confirmed cases was obtained through (1) a basic epidemiological survey performed by 8 public healthcare centers in Daegu, and (2) an in-depth epidemiological survey conducted by the Daegu Center for Infectious Diseases Control and Prevention. Primarily, data from the in-depth epidemiological survey were referenced. When individual data were missing from the in-depth epidemiological survey, data recorded in the basic epidemiological survey were used to supplement the missing information where available.

### Case definition

COVID-19 cases were identified according to the Coronavirus Infectious Disease-19 Response Guidelines of the Korea Centers for Disease Control and Prevention (KCDC) (sixth edition, as of February 20, 2020) [7]. COVID-19 confirmation was determined by real-time reverse transcription polymerase chain reaction assays. Laboratory protocols for the accurate diagnosis of COVID-19 were provided by the Korean Society of Diagnostic Laboratory Medicine and KCDC (second edition, as of February 21, 2020) [8]. The COVID-19 confirmation date for each case was based on the information retrieved from the epidemiological investigation, which was not identical to the confirmation date officially announced by Daegu municipal authorities.

### Study subjects

The study subjects were confirmed COVID-19-positive individuals from February 18, 2020 to June 30, 2020 who participated in the in-depth epidemiological survey in Daegu. There were 7,008 final study subjects. In this study, a member of the S religious group was defined as (1) a case recorded as an S religious group member in the basic epidemiological survey and/or in-depth epidemiological survey, or (2) a case recorded to have attended the worship service of the S religious group in the basic epidemiological survey and/or in-depth epidemiological survey. All other cases were classified as non-members. According to this definition, there were 4,309 members of the S religious group and 2,699 non-members.

### Statistical analysis

The number of confirmed cases per day according to the symptom onset and confirmation date was shown as an epidemic curve. All analysis results were presented by stratifying the cases into S religious group members and non-members. The characteristics of confirmed cases were summarized as frequencies (n) and proportions (%) for categorical variables and means and standard deviations for continuous variables. The case-fatality rate (%) was defined as the number of deaths divided by the number of confirmed cases× 100. The statistical significance of differences in categorical variables between groups was tested using the chisquare test or Fisher exact test. Statistical differences in continuous variables between groups were tested using the independent t-test or Wilcoxon rank-sum test. All statistical analyses were performed using SAS version 9.4 (SAS Institute Inc., Cary, NC, USA). A p-value of < 0.05 was considered to indicate statistical significance.

### Ethics statement

The study protocol was approved by the Institutional Review Board (IRB) of Kyungpook National University Hospital (IRB No. 2020-03-044). The requirement for informed consent was waived by the IRB.

## RESULTS

### The COVID-19 outbreak in Daegu: processes and responses

Table 1 summarizes the processes and responses to the COVID-19 outbreak in Daegu centered on the S religious group after February 18, 2020. The index case of the Daegu COVID-19 outbreak (case #31 in Korea) reported February 7, 2020 as the symptom onset date. A total of 61 cases reported that symptoms appeared before February 7, 2020. Since many confirmed cases were asymptomatic (38.7% in the current study, as discussed below), it was impossible to identify the primary source of infection.

The index case (case #31) attended the worship services of the S religious group on February 9, 2020 and February 16, 2020. Since these dates were within the period of communicability [9], the Daegu church and 24 church-related facilities (including 2 churches, 11 centers, and 11 gospel rooms) of the S religious group immediately closed on February 18, 2020. The Daegu municipal government secured a list of all attendees at the S religious group’s worship services on February 9 and February 16 (n=1,001) and conducted a telephone survey on February 19. It was found that 14.9% of respondents (90/605, corresponding to 9.0% of all attendees) reported symptoms. These 90 symptomatic members were asked to self-isolate and to receive screening tests.

The cumulative number of confirmed cases increased to 108 by February 20, of whom 90 (83.3%) were identified as S religious group members. The Daegu municipal government and health experts determined that COVID-19 had started spreading to the community. All S religious group members were asked to wear face masks indoors and outdoors, as well as to quarantine away from their families. On February 20, a telephone survey was conducted among all members of the S religious group, and self-quarantine was recommended. On February 21, the Daegu government started performing COVID-19 screening tests on 1,261 symptomatic individuals. On February 23, a self-quarantine officer was designated to monitor all members of the S religious group. On February 25, the Daegu government started performing COVID-19 screening tests on asymptomatic individuals from the S religious group and sent a self-quarantine message to all members. Daegu established a 1:3 management system between 3,000 civil servants and 9,336 members of the S religious group, and members’ self-quarantine status was checked twice daily.

On March 2, therapeutic living centers were launched to support patients with mild clinical symptoms and no concurrent medical conditions [10]. All members of the S religious group were advised to undergo COVID-19 screening tests. An administrative order was issued against members who did not undergo screening tests before March 7. As of March 9, 97.6% (10,220/10,471) of the S religious group members had undergone screening tests, and 42.9% (4,137/9,651) of members who were notified of the test results were confirmed as COVID-19 cases [4].

### Distribution of the number of cases per day

**Figure 1.**
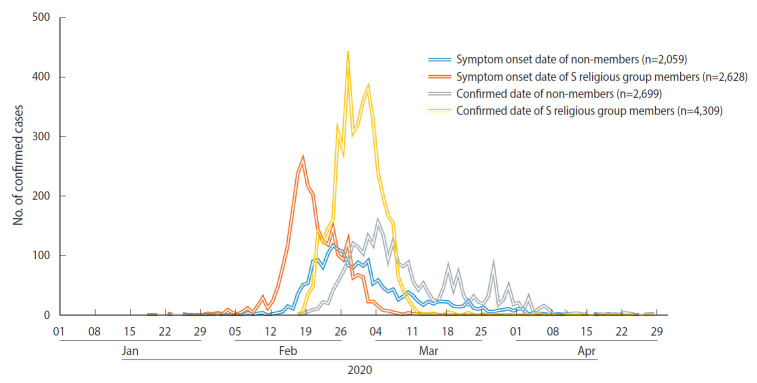
Epidemic curve of coronavirus disease 2019 (COVID-19) in Daegu, Korea, 2020.

The number of confirmed COVID-19 cases in Daegu increased sharply after mid-February and peaked in early March (Figure 1). The curve of the daily number of confirmed cases showed the spread of the epidemic from the S religious group to the general population in the local community. Unlike the general epidemic curve, the S religious group’s epidemic curve was based on active and preemptive screening tests. The number of confirmed cases after early March declined as screening tests were no longer required for the S religious group. However, confirmed cases among non-members continued to increase. Of 7,008 cases, 4,687 (66.9%) reported a symptom onset date during the epidemiological investigation, and 2,321 cases (33.1%) were asymptomatic or had an unclear symptom onset date.

### Characteristics of confirmed COVID-19 cases in Daegu

S religious group members accounted for 61.5% (4,309/7,008) of all confirmed cases in Daegu from February 18, 2020 to June 30, 2020 (Table 2). The worship services attendance rates among confirmed members of the S religious group were 72.8% (3,135/4,309) on February 9, 67.8% (2,921/4,309) on February 12, and 77.4% (3,333/4,309) on February 16. Among all confirmed cases, 5.1% (n=354) lived with a member of the S religious group and 1.5% (n=104) were in contact with a member. Other cases included 19.3% (n=1,355) who had contact with a confirmed case, 0.9% (n=61) who came from overseas, and 11.8% (n=825) with an unknown source of infection at the time of the epidemiological investigation.

Table 3 shows the confirmed cases’ general characteristics according to their membership in the S religious group. The proportion of female among members (66.3%) was higher than among non-members (54.2%, p<0.001). The mean ages of members and non-members were 38.7 years and 53.7 years, respectively (p<0.001). The most represented age groups were 20-29 years (38.3%) among members and 50-59 years (20.9%) among non-members. The comorbid disease prevalence was generally higher in non-members than in members. Hypertension and diabetes mellitus ranked as the first and second most common comorbid diseases in both groups, respectively. The prevalence of hypertension was 4.2% in members and 9.6% in non-members (p<0.001). The prevalence of diabetes mellitus was 2.3% in members and 6.1% in non-members (p<0.001).

### Symptoms of confirmed COVID-19 cases

At the time of the epidemiological investigation, 23.7% of nonmembers and 38.7% of members of the S religious group reported no symptoms (p<0.001) (Table 4). The asymptomatic proportions were higher for members than non-members in the same age groups (Supplementary Material 1). The main symptoms observed in non-members included fever (35.5%), cough (33.7%), myalgia (21.2%), sputum (19.1%), and sore throat (18.9%). Similarly, members experienced main symptoms of cough (25.8%), fever (16.0%), sputum (15.4%), myalgia (14.6%), and sore throat (13.6%). In both groups, less common symptoms included chills, dyspnea, headache, general weakness, rhinorrhea, diarrhea, chest pain or discomfort, a decreased sense of smell and/or taste, dizziness, vomiting, and nausea.

### Case-fatality rate of confirmed COVID-19 cases

The COVID-19 case-fatality rates were 6.4% in non-members and 0.3% in S religious group members (Table 5). In general, the case-fatality rates increased with age. When compared within the age ranges of 60-69 years, 70-79 years, and ≥ 80 years, the case-fatality rates of non-members were significantly higher than those of members within the same age group. The case-fatality rates among cases > 80 years old were 31.2% in non-members and 7.7% in members (p=0.012). Compared with the case-fatality rate of all confirmed cases in Korea until early February 2021 [11], the case-fatality rate of non-members was higher, and that of members was lower.

### Examples of non-cooperation with countermeasures of quarantine authorities

According to the regular briefings by the Daegu municipal government on the COVID-19 response [4], there were several examples of individual members or executives of the S religious group interfering with the city’s countermeasures. First, the executives of the S religious group submitted a membership list that omitted some members. Second, some cases were concealed or misrepresented in matters related to the religion during the epidemiological investigations, confusing the quarantine authorities’ responses (14th regular briefing report of Daegu, February 28, 2020). Third, there were cases of refusal of screening tests, violation of self-isolation guidelines, and refusal to transfer to a hospital or therapeutic living center (16th report briefing report of Daegu, March 1, 2020). The reasons for refusing to enter the therapeutic living center included wanting to stay with their children and families or a request for hospital admission (25th report briefing report of Daegu, March 10, 2020).

Compared to non-members, the intervals from symptom onset to diagnosis (p<0.001) and from diagnosis to admission (p<0.001) of members were longer, whereas the interval from admission to discharge (p<0.001) was shorter during the same period (Supplementary Material 2).

## DISCUSSION

The COVID-19 outbreak in Daegu started with identifying case #31 in Korea on February 18, 2020. This case was a member of the S religious group, and she had attended worship services with hundreds of members. By conducting preemptive screening tests for all S religious group members within a short period, the number of COVID-19 cases increased sharply. The COVID-19 outbreak triggered by religious activities resulted in rapid transmission of the virus to high-risk groups in the community. Compared with non-members, the members of the S religious group had a higher proportion of female, a younger average age, and a lower prevalence of underlying diseases.

The proportion of asymptomatic COVID-19 infections is estimated to be 18% to 81% [12]. Recent studies reported that symptomatic COVID-19 infections were positively correlated with older age, working outside the home, respiratory comorbidities, and alcohol use [13,14]. The proportion of asymptomatic cases in our study at the time of the epidemiological investigation was higher among members than non-members. Symptom onset of cases may have occurred after the epidemiological investigation, although we were unable to follow-up whether they developed symptoms after self-isolation or hospital admission. Therefore, the asymptomatic proportion of either group may have been overestimated. In another study of 10 asymptomatic COVID-19 cases at diagnosis, 6 cases reported symptoms during a follow-up and in-depth interview [15].

When compared within the same age groups, the proportions of asymptomatic cases among S religious group members were lower than those among non-members (Supplementary Material 1). Early extensive screening tests performed on > 10,000 members may have contributed to the high proportion of asymptomatic or mild infections. Considering the example of providing inaccurate information to the Daegu municipal government, the possibility of under-reporting symptoms cannot be excluded. Because the proportion of asymptomatic cases may be high in the early stages of COVID-19 infection, public health measures to block silent infections in the early period of an outbreak in the community are essential.

In our study, the case-fatality rate was higher in non-members than in members of the S religious group. This finding can be attributed to the older age distribution of non-members compared with members. However, a comparison within the same age groups showed that the case-fatality rates of non-members were higher than those of members. This may have been because many non-members had underlying diseases and were hospitalized in long-term care or psychiatric hospitals [16]. The low case fatality observed among members may suggest a kind of “healthy worker effect,” considering that the case-fatality rates of the S religious group members were lower than those of all cases in Korea in the same age groups. People with serious illnesses are less likely to engage in external religious activities [17]. Those who have better overall health are likely to participate in energetic religious activities. Positive effects of religious activity on health have also been reported [18,19].

The S religious group is derived from the Christian new religious movement in Korea. The unusual religious practices of the S religious group seem to have provided ideal conditions for COVID-19 to rapidly spread among members. Since the activities of the S religious group are often conducted in secret and there is little information regarding them available to the public, it is challenging to present qualitative and quantitative data on their religious activities. Close contact between members in a confined space during religious events (e.g., worship service) may result in wide spread of infectious diseases. It is known that hundreds of members sit side by side in rows on the floor to worship in groups. A high-density work environment can also be a high-risk factor for the transmission of infectious diseases. COVID-19 outbreaks in crowded office settings, such as call centers, have been reported [20]. In religious groups, singing or speaking out loud during hymns and vocal prayers in church services can contribute to the rapid spread of COVID-19. Starting on July 10, 2020, the Korean government mandated churches to comply with core quarantine rules. These included keeping at least a 1 m distance between churchgoers (ideally 2 m), prohibiting hymns and vocal prayers during church services, and prohibiting the consumption of food in the facility [21].

Members of the S religious group are also known to lead special group lives. Some live in certain areas or within a single household. During the COVID-19 outbreak, it was discovered that many confirmed cases occurred in the H apartment buildings in Daegu [4]. All 46 confirmed cases in the apartment buildings were members of the S religious group. This outbreak in the apartment buildings served as a motivation to identify the group residence behavior of the religious group. An additional investigation on the residential facilities of religious groups by the Daegu municipal government identified 64 residential complexes in which ≥ 10 members resided (26th regular briefing report of Daegu, March 11, 2020). Due to concern regarding contact with the fully recovered members of the S religious group, all confirmed members who were in self-isolation at home were forcibly admitted to a hospital or therapeutic living center.

The current study has some limitations. First, since the epidemiological investigation was not prepared for research, some variables were absent or insufficient (e.g., clinical severity). Second, because the epidemiological investigation was conducted during the rapid increase in the number of confirmed cases, some information may have been collected incorrectly; however, no further investigation could be done. For example, a confirmed case reported January 1, 2020 as the symptom onset date (for reference, the Daegu COVID-19 outbreak was detected on February 18, 2020). It would be necessary to confirm whether the questionnaire was filled out correctly or if the respondent understood and answered the question accurately, but it was impossible to do so. Caution should be taken when interpreting these results because the values of the epidemiological investigation variables (including asymptomatic proportion) may change according to a further in-depth investigation. In addition, some cases’ responses were difficult to classify into symptoms or diseases using appropriate medical terminology because some cases responded in plain language or used incorrect medical terms. A standardized epidemiological investigation form and guidelines are needed to collect systematic and efficient information. To conduct statistical analyses using epidemiological investigation data surveyed on paper, it took a long time to code and clean the data. If a system that can use computer-based or web-based questionnaires is built, the time required to analyze epidemiological data will be reduced. Third, S religious group members who were confirmed after the end of June or that lived outside Daegu were not included in the study. However, this study can be regarded as providing representative data for the S religious group, since our study included > 80% of confirmed cases who were S religious group members. The total number of confirmed COVID-19 cases in the S religious group was 5,214 (4,512 in Daegu and 702 in other regions), accounting for 6.4% of all confirmed cases (n=81,487) in Korea as of February 9, 2021 [11].

In this study, major epidemiological indicators, such as the exposure date, incubation period, and COVID-19 generation time, were not evaluated. In the S religious group, there was a large-scale epidemic within a short period of time. Most of the members attended worship services several times, often participated in religious activities other than worship, and frequently lived in the same household. Thus, we judged that estimations of individual exposure dates would be inaccurate. For the same reason, it was impossible to distinguish between the nth generation of infection among confirmed cases. In addition, from the early days of the outbreak, epidemiological investigations focused on identifying the daily movements (visited places) of confirmed cases. In the in-depth epidemiological survey, the visited places of confirmed cases were recorded in detail. However, information on individuals they were in contact with and the associated contact dates was not recorded systematically in many cases. Combining additional data and analyses in the future will allow major epidemiological indicators to be evaluated. In addition, useful information on COVID-19 can be obtained in the future if the confirmed case data can be linked with global positioning system or drug utilization review data.

The main strength of this study is that it examined the COVID-19 outbreak triggered by the special circumstances of religious activities in the local community. Moreover, it summarized the initial characteristics in a large sample size of confirmed cases in the early stages of the COVID-19 outbreak according to S religious group membership and the Daegu municipal government’s response to the outbreak. Compared with a study that reported the characteristics of cases who received treatment after admission to the hospital [5], the characteristics such as the distribution of age, prevalence of comorbid diseases, and proportion of asymptomatic infections were different.

In conclusion, the Daegu COVID-19 outbreak in February 2020 was mainly caused by a religious group’s activities. It was impossible to identify the first case of infection due to the high proportion of asymptomatic cases. The confirmed cases who were members of the S religious group showed different epidemiological characteristics from the non-member cases. Countermeasures to separate S religious group members from other residents, including isolation and screening tests for all members, prevented the further massive spread of COVID-19 in the community.

## Figures and Tables

**Table 1.** Summary of the processes and responses centered on the S religious group during the coronavirus disease 2019 (COVID-19) outbreak in Daegu, Korea, 2020

Date in 2020	Cumulative no. of confirmed cases^1^		Key countermeasures
	Members of the S religious group	Total	
Feb 18	7	10	Index case (case #31 in Korea) confirmed
			Forced closure of church and 24 related facilities of the S religious group
Feb 19	42	51	Securing a list of members
			Telephone survey of symptoms from worship attendees (n=1,001)
			Request of self-quarantine and screening test for 90 symptomatic persons
Feb 20	90	108	Telephone survey of symptoms from all members (n=9,336)
			Decision of self-quarantine for all members
Feb 21	226	255	Start of COVID-19 screening test for 1,261 symptomatic persons
Feb 23	494	565	Designation of a self-isolation monitoring officer
Feb 25	962	1,130	Start of COVID-19 screening test for non-symptomatic persons
			A message sent for self-quarantine to all members
Feb 26	1,241	1,480	Executive order for church-related facilities closure
			Securing additional list of members
Feb 29	2,292	2,856	Securing the list of disciples
			Extension of the period of self-quarantine to the unexamined members
Mar 2	3,035	3,834	Opening of the first therapeutic living center
Mar 4	3,603	4,676	Outbreak in the H apartment buildings where members resided in groups
Mar 7	4,124	5,547	Executive order to screen unexamined members
Mar 10	4,254	5,932	Decision to force all confirmed patients in homes to be admitted to a facility
Mar 11	4,267	6,001	Completion of the screening test for all members
Jun 30	4,309	7,008	Overall, 7,008 COVID-19 cases were confirmed

1 The figures are based on the COVID-19 confirmed date of the epidemiological investigation reports, which differed from the confirmed date officially announced by the Daegu municipal government.

**Table 2.** Presumed modes of coronavirus disease 2019 (COVID-19) transmission in Daegu, Korea (n=7,008)

Mode of transmission	n (%)
Member of the S religious group	4,309 (61.5)
Cohabitation with a member of the S religious group	354 (5.1)
Contact with a member of the S religious group	104 (1.5)
Contact with a confirmed case	1,355 (19.3)
Overseas	61 (0.9)
Unknown	825 (11.8)

**Table 3.** Characteristics of confirmed COVID-19 cases according to membership in the S religious group in Daegu, Korea (n=7,008)

Characteristics	Membership in the S religious group		p-value^1^
	Non-members (n=2,699)	Members (n=4,309)	
Sex			
Male	1,235 (45.8)	1,453 (33.7)	<0.001
Female	1,464 (54.2)	2,856 (66.3)	
Nationality			
Korean	2,656 (98.4)	4,281 (99.4)	<0.001
Foreign	43 (1.6)	28 (0.7)	
Place of residence			
Daegu	2,687 (99.6)	4,309 (100)	<0.001
Gyeongsangbuk-do	3 (0.1)	0 (0.0)	
Others/unknown	9 (0.3)	0 (0.0)	
Age (yr)			
Mean±SD	53.7±20.5	38.7±16.9	<0.001
0-9	56 (2.1)	21 (0.5)	<0.001
10-19	120 (4.5)	255 (5.9)	
20-29	267 (9.9)	1,652 (38.3)	
30-39	198 (7.3)	465 (10.8)	
40-49	343 (12.7)	574 (13.3)	
50-59	564 (20.9)	758 (17.6)	
60-69	518 (19.2)	412 (9.6)	
70-79	367 (13.6)	146 (3.4)	
≥80	266 (9.9)	26 (0.6)	
Comorbidity			
Hypertension	258 (9.6)	182 (4.2)	<0.001
Diabetes mellitus	165 (6.1)	100 (2.3)	<0.001
Cancer	44 (1.6)	27 (0.6)	<0.001
Chronic renal failure	11 (0.4)	1 (0.0)	<0.001
COPD	5 (0.2)	0 (0.0)	0.009
Asthma	29 (1.1)	31 (0.7)	0.116
Heart disease	60 (2.2)	31 (0.7)	<0.001
Stroke	32 (1.2)	18 (0.4)	<0.001
Dementia	31 (1.1)	3 (0.1)	<0.001

Values are presented as number (%). COVID-19, coronavirus disease 2019; SD, standard deviation; COPD, chronic obstructive pulmonary disease.

1 Chi-square test or Fisher exact test for categorical variables and independent t-test for continuous variables.

**Table 4.** Symptoms at the time of the epidemiological investigation of confirmed coronavirus disease 2019 (COVID-19) cases according to membership in the S religious group in Daegu, Korea (n=7,008)

Symptoms	Membership in the S religious group		p-value^1^
	Non-members (n=2,699)	Members (n=4,309)	
Presence of symptoms			
Asymptomatic	639 (23.7)	1,669 (38.7)	<0.001
Symptomatic	2,060 (76.3)	2,640 (61.3)	
Symptoms			
Fever/febrile sense	957 (35.5)	691 (16.0)	<0.001
Cough	910 (33.7)	1,111 (25.8)	<0.001
Myalgia	571 (21.2)	631 (14.6)	<0.001
Sputum	516 (19.1)	663 (15.4)	<0.001
Sore throat	511 (18.9)	585 (13.6)	<0.001
Chills	391 (14.5)	377 (8.8)	<0.001
Dyspnea	205 (7.6)	149 (3.5)	<0.001
Headache	196 (7.3)	296 (6.9)	0.531
General weakness/general ache	175 (6.5)	231 (5.4)	0.050
Rhinorrhea	158 (5.9)	358 (8.3)	<0.001
Diarrhea	62 (2.3)	48 (1.1)	<0.001
Chest pain/chest discomfort	41 (1.5)	41 (1.0)	0.032
Decreased sense of smell	32 (1.2)	67 (1.6)	0.202
Decreased sense of taste	27 (1.0)	37 (0.9)	0.544
Dizziness	21 (0.8)	25 (0.6)	0.318
Vomiting	20 (0.7)	5 (0.1)	<0.001
Nausea	17 (0.6)	15 (0.3)	0.089
Other	102 (3.8)	70 (1.6)	<0.001

Values are presented as number (%).

1 Chi-square test or Fisher exact test.

**Table 5.** Case-fatality rate of confirmed COVID-19 cases according to membership in the S religious group in Daegu, Korea (n=7,008)

Characteristics	Membership in the S religious group				p-value^1^	Korea^2^(n=81,487)
	Non-members (n=2,699)		Members (n=4,309)			
	No. of deaths/cases	Case-fatality rate, %	No. of deaths/cases	Case-fatality rate, %		Case-fatality rate, %
Total	173/2,699	6.4	12/4,309	0.3	<0.001	1.82
Sex						
Male	87/1,235	7.0	9/1,453	0.6	<0.001	1.84
Female	86/1,464	5.9	3/2,856	0.1	<0.001	1.80
p-value	0.216		0.004			
Age (yr)						
0-9	0/56	0.0	0/21	0.0	NA	0.00
10-19	0/120	0.0	0/255	0.0	NA	0.00
20-29	0/267	0.0	0/1,652	0.0	NA	0.00
30-39	1/198	0.5	0/465	0.0	0.299	0.06
40-49	1/343	0.3	0/574	0.0	0.374	0.10
50-59	5/564	0.9	4/758	0.5	0.508	0.32
60-69	28/518	5.4	1/412	0.2	<0.001	1.33
70-79	55/367	15.0	5/146	3.4	<0.001	6.47
≥80	83/266	31.2	2/26	7.7	0.012	20.74
p-value	<0.001		<0.001			

COVID-19, coronavirus disease 2019; NA, not applicable.

1 Chi-square test or Fisher exact test for a difference between groups.

2 Source from: Korea Centers for Disease Control and Prevention. Updates on COVID-19 in Republic of Korea [11].
