# Supplementary material for: Epidemiological characteristics of a COVID-19 outbreak caused by religious activities in Daegu, Korea
Source: Epidemiol Health. 2021 Apr 14;43:e2021024. doi: 10.4178/epih.e2021024 (PMC8189844; doi:10.4178/epih.e2021024)
Supplement: Supplementary file 1 [file epih-43-e2021024-suppl.docx]

Supplementary Material 1. Asymptomatic proportion of COVID-19 cases according to the age group in Daegu, Korea (n=7,008)

| **Characteristics** | **Membership in the S religious group** | | | | |  |
| --- | --- | --- | --- | --- | --- | --- |
|  | Non-members  (n=2,699) | | Members  (n=4,309) | |  | |
|  | n | (%) | n | (%) | p-value^1^ | |
| Total | 639/2,699 | (23.7) | 1,669/4,309 | (38.7) | <0.001 | |
| Age, years |  |  |  |  |  | |
| 0–9 | 17/56 | (30.4) | 10/21 | (47.6) | 0.157 | |
| 10–19 | 34/120 | (28.3) | 134/255 | (52.6) | <0.001 | |
| 20–29 | 54/267 | (20.2) | 670/1,652 | (40.6) | <0.001 | |
| 30–39 | 42/198 | (21.2) | 166/465 | (35.7) | <0.001 | |
| 40–49 | 75/343 | (21.9) | 198/574 | (34.5) | <0.001 | |
| 50–59 | 137/564 | (24.3) | 271/758 | (35.8) | <0.001 | |
| 60–69 | 122/518 | (23.6) | 150/412 | (36.4) | <0.001 | |
| 70–79 | 71/367 | (19.4) | 59/146 | (40.4) | <0.001 | |
| ≥80 | 87/266 | (32.7) | 11/26 | (42.3) | 0.322 | |
|  | p-value | 0.005 | p-value | <0.001 |  | |

COVID-19, coronavirus disease 2019.
^1^Chi-square test or Fisher exact test for a difference between groups.

Supplementary Material 2. Epidemiologic indicators among confirmed COVID-19 cases according to the membership in the S religious group in Daegu, Korea (n=7,008)

|  | Membership in the S religious group | | | |  |
| --- | --- | --- | --- | --- | --- |
| Indicators^1^  Period divided by confirmed date | Non-members  (n=2,699) | | Members  (n=4,309) | |  |
|  | n | Mean ± SD (median) | n | Mean ± SD (median) | p-value^2^ |
| Symptom-to-diagnosis (days)^3^ | 1,663 | 6.5 ± 5.3 (5) | 2,387 | 7.6 ± 5.7 (7) | <0.001 |
| February 18 – February 22 | 42 | 3.0 ± 2.7 (2) | 310 | 4.6 ± 3.8 (4) | <0.001 |
| February 23 – February 27 | 231 | 4.7 ± 3.6 (4) | 962 | 6.8 ± 4.9 (6.5) | <0.001 |
| February 28 – March 3 | 456 | 6.2 ± 4.5 (5) | 798 | 8.5 ± 5.7 (8) | <0.001 |
| March 4 – March 8 | 449 | 7.2 ± 4.9 (6) | 276 | 10.7 ± 7.4 (9) | <0.001 |
| March 9 – | 485 | 7.3 ± 6.7 (5) | 41 | 11.5 ± 8.7 (12) | 0.058 |
| Diagnosis-to-admission (days) | 1,923 | 3.8 ± 3.8 (3) | 3,994 | 5.6 ± 3.7 (5) | <0.001 |
| February 18 – February 22 | 40 | 4.4 ± 4.5 (2) | 313 | 4.7 ± 4.9 (2) | 0.557 |
| February 23 – February 27 | 243 | 4.8 ± 3.9 (4) | 1,188 | 6.3 ± 4.0 (6) | <0.001 |
| February 28 – March 3 | 492 | 5.1 ± 4.5 (4) | 1,621 | 6.0 ± 3.4 (6) | <0.001 |
| March 4 – March 8 | 505 | 4.3 ± 3.2 (4) | 770 | 4.4 ± 2.8 (4) | 0.003 |
| March 9 – | 643 | 1.9 ± 2.6 (1) | 102 | 2.3 ± 1.8 (2) | 0.008 |
| Admission-to-discharge (days) | 1,886 | 23.4 ± 13.5 (21) | 3,997 | 20.5 ± 12.7 (17) | <0.001 |
| February 18 – February 22 | 39 | 22.7 ± 11.9 (20) | 311 | 26.7 ± 15.1 (24) | 0.234 |
| February 23 – February 27 | 234 | 24.9 ± 13.4 (23) | 1,185 | 21.8 ± 13.0 (19) | <0.001 |
| February 28 – March 3 | 470 | 23.6 ± 13.7 (21) | 1,624 | 19.1 ± 11.8 (17) | <0.001 |
| March 4 – March 8 | 505 | 24.2 ± 14.3 (21) | 772 | 19.5 ± 11.9 (17) | <0.001 |
| March 9 – | 638 | 22.2 ± 12.8 (19) | 105 | 17.2 ± 11.8 (16) | <0.001 |
| Symptom to Death (days) | 146 | 24.0 ± 19.6 (18.5) | 10 | 25.3 ± 15.1 (25) | 0.543 |
| Diagnosis to Death (days) | 173 | 18.2 ± 17.9 (12) | 12 | 24.3 ± 20.9 (19.5) | 0.317 |

COVID-19, coronavirus disease 2019; SD: standard deviation

^1^ Symptom-to-diagnosis, the period from symptom onset to diagnosis; Diagnosis-to-admission, the period from diagnosis to admission to hospital or therapeutic living center; and Diagnosis-to-admission, the period from admission to hospital or therapeutic living center. ^2^ Independent t-test or Wilcoxon rank-sum test. ^3^calculated after excluding cases with symptoms after COVID-19 confirmation (18 cases among non-members, 30 cases among members).
